# Quantitative Comparison of Conventional and t-SNE-guided Gating Analyses

**DOI:** 10.3389/fimmu.2019.01194

**Published:** 2019-06-05

**Authors:** Shadi Toghi Eshghi, Amelia Au-Yeung, Chikara Takahashi, Christopher R. Bolen, Maclean N. Nyachienga, Sean P. Lear, Cherie Green, W. Rodney Mathews, William E. O'Gorman

**Affiliations:** ^1^OMNI Biomarker Development, Genentech Inc., South San Francisco, CA, United States; ^2^Bioinformatics, Genentech Inc., South San Francisco, CA, United States

**Keywords:** cyTOF, t-SNE, cytometry informatics, dimensionality reduction, immunophenotyping, high-dimensional cytometry

## Abstract

Dimensionality reduction using the t-Distributed Stochastic Neighbor Embedding (t-SNE) algorithm has emerged as a popular tool for visualizing high-parameter single-cell data. While this approach has obvious potential for data visualization it remains unclear how t-SNE analysis compares to conventional manual hand-gating in stratifying and quantitating the frequency of diverse immune cell populations. We applied a comprehensive 38-parameter mass cytometry panel to human blood and compared the frequencies of 28 immune cell subsets using both conventional bivariate and t-SNE-guided manual gating. t-SNE analysis was capable of stratifying every general cellular lineage and most sub-lineages with high correlation between conventional and t-SNE-guided cell frequency calculations. However, specific immune cell subsets delineated by the manual gating of continuous variables were not fully separated in t-SNE space thus causing discrepancies in subset identification and quantification between these analytical approaches. Overall, these studies highlight the consistency between t-SNE and conventional hand-gating in stratifying general immune cell lineages while demonstrating that particular cell subsets defined by conventional manual gating may be intermingled in t-SNE space.

## Introduction

The analysis of cytometry data through manual “hand-gating” has progressively become more and more impractical as cytometry data sets continue to increase in dimensionality and size (1). The sequential inspection and gating of more than 20 bivariate plots is now necessary to conduct even a basic immunophenotyping analysis of 40-parameter data. The primary problems with conventional cytometric analysis are the subjectivity of operator-defined gating thresholds and the low throughput of manual gating (2, 3). Frequency quantitation of cell subsets defined by the subjective discretization of continuously distributed markers, such as CCR7 and CD45RA in defining T cell subsets, are particularly subject to inter-analyst variability. The goal of cytometry informatics is to automate, or at least augment, the objective stratification of cell populations in cytometry data sets. Although an overwhelming variety of computational tools have been developed as potential alternatives to manual hand-gated analyses (4), the field has yet to unite around a single computational approach.

Clustering and dimensionality reduction are algorithmic methods that have frequently been applied to cytometry data. Over the past decade, clustering algorithms have been critically assessed through comparison to expert manual analysis as well as through the cross-validation of clustering results between different clustering algorithms (5, 6). In contrast, there have been very few critical assessments of dimensionality reduction as a cytometric analytical tool or even as a tool that simply enables the visualization of single-cell data (7, 8).

Recently, the dimensionality reduction algorithm, t-distributed stochastic neighbor embedding (t-SNE) (9), has gained popularity as a means to visualize high dimensional single-cell data (10–12). While t-SNE produces bivariate dot-plot based visualizations that are inherently intuitive for cytometrists to comprehend, there is still an important need to assess the strengths and limitations of this approach, especially in respect to how t-SNE relates to expert manual hand-gated analysis which has historically been the gold standard.

Currently, in cytometric analysis t-SNE is typically used as a visualization tool to qualitatively assess cell population diversity, rather than as a quantitative analytical tool for calculating the frequency of specific cell populations. In these studies, we sought to quantitatively compare cell population frequencies determined by both conventional bivariate plot-based and t-SNE-guided manual gating. Our goal was not to definitively validate dimensionality reduction as a quantitative analytical approach, but simply to understand the relationship between dimensionality reduction and conventional manual gating in defining canonical cell populations. Given that t-SNE analysis is primarily being used to explore novel cellular landscapes, the ability of this approach to accurately represent well-characterized and defined populations is important for establishing its general validity. Alternatively, t-SNE mapping could reveal flaws in conventional gating strategies.

We found that immune populations stratified by divergent and discrete marker expression in a conventional analysis were also well separated by t-SNE dimensionality reduction. As expected, the projection of general cell lineages identified via conventional gating onto the t-SNE map demonstrated congruence in the cell populations distinguished by these analytical approaches. In contrast, particular T cell subsets defined by continuous markers were often not well separated in t-SNE space. In these cases, the projection of hand-gated T cell populations onto the t-SNE map showed high levels of interspersion between subsets. Isolation and t-SNE analysis of only the CD4+ T cell lineage produced only marginally better separation between canonical subsets than in the global analysis. In summary, cell populations that are well stratified by conventional bivariate plot-based gating will also be separated via t-SNE-based dimensionality reduction; however, subsets defined by the gating of continuous markers on a bivariate plot will not be fully separated in t-SNE space unless discrete orthogonal markers are included that facilitate further stratification.

## Materials and Methods

### PBMC Isolation

Healthy human donors (*N* = 10) peripheral blood mononuclear cells (PBMCs) were isolated using 50 mL Leucosep™ tubes (Greiner Bio-One International, Germany) and Ficoll-Paque™ PLUS (GE Healthcare, Sweden). Whole blood was drawn into sodium heparin anticoagulant collection tubes and diluted with phosphate-buffered saline (PBS) without calcium or magnesium (Lonza, Walkersville, Maryland). Whole blood was centrifuged for 15 min at 800x g at room temperature (RT). PBMCs were then harvested and washed with PBS and centrifuged for 10 min at 250x g at RT before preparation for cell staining.

### Source of mAb-Isotope Conjugates

See Table S1 for a list of the metal conjugated mAbs used in these studies. In-house conjugations were performed using Multi-Metal Maxpar® Kits (Fluidigm, South San Francisco, California). 115In was purchased from Trace Sciences International Corporation.

### Staining of Cells

Washed PBMCs were re-suspended at a cell concentration of 10^7^ cells/mL with PBS. Cells were then incubated with a viability reagent, Cell-ID™ Cisplatin (Fluidigm, South San Francisco, California) at a final concentration of 5 μM for 5 min on ice. Viability staining was quenched with a 5x volume of MaxPar® Cell Staining Buffer (Fluidigm, South San Francisco, California) and centrifuged at 300x g, then re-suspended to a final concentration of 30 million cells/mL in staining buffer. For antibody labeling, 3 million cells were transferred to Falcon® 5 mL 12 × 75 mm tubes (Corning, New York). To block Fc receptor binding, cells were incubated with 5 μL of Human TruStain FcX™ (BioLegend, San Diego, California) for 10 min on ice. A master mAb cocktail containing all metal-conjugated surface antibodies (50 μL of total staining reagent volume) was added to samples for cell-surface staining and incubated for 30 min on ice. See Table S1 for a list of the metal conjugated mAbs used in these studies. Cells were then washed once with 4 mL cell staining buffer to prepare for intracellular staining. Briefly, cells were re-suspended in 1 mL of fixation/permeabilization solution by using the FoxP3 Staining Buffer Set (eBioscience, San Diego, California) for 45 min on ice, washed with 3 mL of permeabilization buffer at 800x g for 5 min, and re-suspended in 50 μL of permeabilization buffer. Cells were then stained for intracellular targets by addition of 50 μL antibody cocktail. After 30 min incubation on ice, the cells were washed with 4 mL cell staining buffer and fixed overnight at 4°C in a 1 mL solution containing Cell-ID™ Intercalator-Ir in 1.6% EMS Fix (Electron Microscopy Sciences, Hatfield, Pennsylvania).

For flow cytometry, 20 μL of the TBNK cocktail from BD Biosciences was added into each of the 10 TruCount FACS tubes. 100 μL of each donor's blood was reverse pipetted into the TruCount tubes and incubated for 30 min. After incubation, 450 μL of 1x BD FACS Lysing solution was added and incubated for 15 min. Samples were then acquired within an hour from lysing on a FACS Canto II (BD Biosciences, San Jose, CA).

### Acquisition on CyTOF® Instrument

Cells were washed with 3 mL of MaxPar® cell staining buffer and centrifugated at 800x g for 5 min followed by a 2x wash with 4 mL MaxPar® Water (Fluidigm, South San Francisco, California). Before introduction into the Helios™, a CyTOF® System (Fluidigm, South San Francisco, California), pelleted cells were re-suspended with MaxPar® Water containing EQ™ Four Element Calibration Beads (Fluidigm, South San Francisco, California) and filtered using a 12 × 75 mm tube with a 35 μm nylon mesh cell-strainer cap (Corning, New York).

### Data Processing and Analysis

All FCS files were normalized using the MATLAB® (MathWorks®, Natick, Massachusetts) normalizer and analyzed using FlowJo® software (Flowjo, Ashland, Oregon).

### Dimensionality Reduction (t-SNE) Analysis

Individual donor fcs files were imported into R and their expression matrices containing measured intensities for each marker at single-cell level were extracted using functions from the flowCore package (13). A subset of 50,000 cells were selected for each donor at random and merged into a single expression matrix prior to t-SNE analysis. The following channels were removed from the expression matrix to only include protein markers in t-SNE analysis: beads, event length, intercalator, viability, center, offset, residual, and time. Marker intensities were transformed using the inverse hyperbolic sine (arcsinh) function. A total of 500,000 cells and 38 markers (Table S1) were used to create a t-SNE map of the peripheral human immune system.

The Barnes-Hut implementation of t-SNE by the Rtsne package (14) with 1,000 iterations, a perplexity parameter of 30, and a trade-off θ of 0.5 (9, 15), was used for applying the dimensionality reduction algorithm. The output was in the form of a matrix with 500,000 rows and 2 columns corresponding to t-SNE dimension 1 and dimension 2. t-SNE maps were generated by plotting each event by its t-SNE dimensions in a dot-plot. Intensities for markers of interest were overlaid on the dot-plot to show the expression of those markers on different cell islands and facilitate assignment of cell subsets to these islands. The t-SNE dimensions were appended to the original expression matrix as derived parameters and exported as an fcs file, which could subsequently be opened and analyzed using FlowJo (Ashland, Oregon). For flow cytometry data FCS Express (Glendale, California) was used to conduct t-SNE analysis.

To evaluate the impact of modulating pre-specified parameters on t-SNE map generation, a subset of the 10-donor data (50,000 cells) was analyzed using varying perplexity, iteration number and trade-off θ values. Perplexity of 5, 30, and 100, iteration number of 1,000 and 10,000 and trade-off θ of 0.2, 0.5, and 0.8 were compared. The impact of limiting the markers used to construct a global t-SNE map to only general lineage markers was examined by running t-SNE with only the following markers: CD45, CD3, CD4, CD8, γδTCR, IgD, CD19, CD20, HLA-DR, CD14, CD11c, CD66, CD56, CD16, CD1c, CD38, and CD11b (data not shown).

Concordance between manually hand gated populations projected onto t-SNE space and computationally defined clusters was qualitatively assessed for Phenograph (16), DensVM (17), and FlowSOM (18) clustering methods. Clustering and overlay with t-SNE maps was performed using the cytofkit package in R (19).

### Comparison of Hand-gated and t-SNE-guided Gated Subsets at Single-cell Level

To evaluate the concordance between hand-gating and t-SNE-guided gating at single-cell level, the FlowJo workspace file (tsp) of the aggregated and t-SNE appended fcs file was imported into R. For each population, the cells captured in the corresponding subset using hand-gating or t-SNE-guided gating were extracted and compared between the two methods. t-SNE-guided manual gates were drawn based on observed boundaries of canonical phenotypic marker expression rather than on cell subset density. The ability of the t-SNE-guided gating to match the hand-gating results was quantified by the fraction of cells in the hand-gated population that matched with the t-SNE-guided population. This was calculated by dividing the number of cells in the overlap between the two gates by the total number of cells in the hand-gated population. The fraction of cells in the t-SNE-guided gated population that matched with the hand-gated population was similarly calculated by dividing the number of cells in the overlap between the gates by the total number of cells in the t-SNE-guided gate (Supporting Information Figure S5). The flowWorkspace package (2) was used for analysis of wsp file in R.

## Results

### Conventional Bivariate Plot-based Gating Strategy Defining the Peripheral Human Immune System

**Figure 1:**
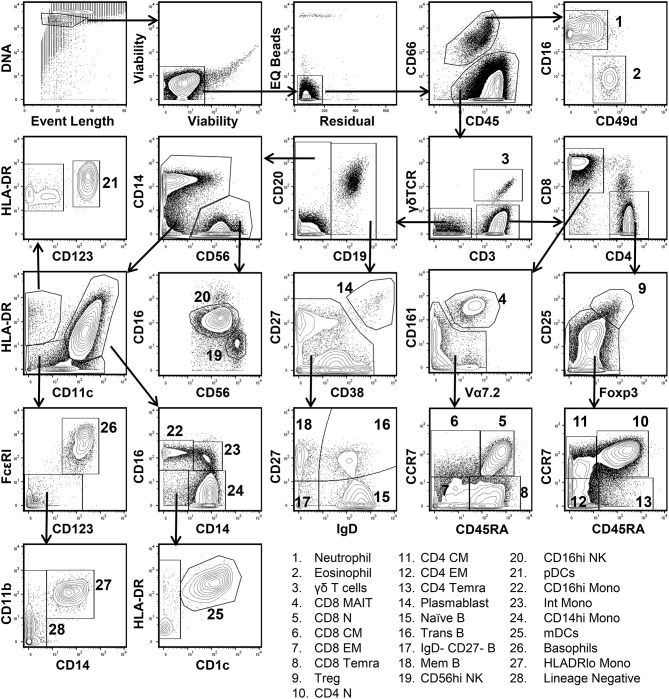
Conventional manual gating strategy for 38-parameter human immunophenotyping. Human PBMCs were isolated and prepared for mass cytometry analysis as described in Materials and Methods section. Data shown are from the merger of 10 donor samples (50,000 cells per donor) into a single fcs file. Single living cells were identified based on intercalator 193Ir, event length, cisplatin 192 & 195Pt, EQ Bead (140Ce), and residual signal intensity. Differential expression of CD66, CD16, and CD49d was used to discern Neutrophils and Eosinophils from other immune cell populations. CD3, CD4, CD8, CD25, CD45RA, CD161, Vα7.2, CCR7, Foxp3, and γδ-TCR expression were used to define 11 T cell subsets. CD19, CD20, CD27, CD38, and IgD were used to define 5 B cell subsets. Two NK cell subsets were defined based on CD56 and CD16 expression. Plasmacytoid dendritic cells (pDCs) were CD11clo, HLADRhi, and expressed high levels of CD123. Monocyte and myeloid dendritic cell subsets (mDCs) were identified based on the differential expression of HLADR, CD11c, CD14, CD16, CD11b, and CD1c. Basophils co-express high levels of CD123 and FcεR1.

In order to define the relationship between cell populations stratified by a global t-SNE map of the peripheral immune system and a conventional hand-gating analysis, a diverse 38-parameter cytometry panel was applied to identify 28 distinct immune cell populations in human blood (Table S1). Peripheral blood mononuclear cells (PBMCs) from 10 healthy donors were isolated and processed for mass cytometric analysis. Figure 1 shows the conventional manual gating strategy used to define these populations. Neutrophils and Eosinophils were identified based on CD66 positivity and further subsetted based on CD16 and CD49d expression (20). CD3, CD4, CD8, CCR7, CD45RA, CD25, and Foxp3 were used to identify regulatory, naïve, central, effector, and effector memory T cells expressing CD45RA (TEMRA) (21). γδ TCR, Vα7.2 TCR, and CD161 expression defined γδ and mucosal associated invariant TCR (MAIT) T cells (22). CD19+ B cell subsets were defined based on the differential expression of CD27, CD38, and IgD into plasmablast, naïve, memory, transitional, and double-negative subsets (23). Following the exclusion of the lineages described above, two NK cell populations were identified based on the expression of CD56 and CD16 (24). Monocytes and dendritic cell subsets were identified based on the differential expression of CD11c, CD11b, HLADR, CD14, CD1c, and CD123 (25). Lastly, FcεRI and CD123 co-expression identified basophils.

### Comparison of Conventional and t-SNE-guided Manual Analysis Across General Immune Cell Lineages

For t-SNE analysis singlet and viability gating was performed manually prior to data export for downstream computation (see Figure S1 for a workflow schematic and Materials and Methods section for details on t-SNE analysis). Due to the stochastic nature of t-SNE, analyzing each donor independently would yield maps in which the same cell populations would be in different locations. Data merger was therefore conducted to ensure spatial alignment of the same cell populations between donors in t-SNE space. 50,000 cells were randomly sampled per donor to create a final file containing 500,000 total cells. Specific donor identifiers were integrated prior to data merger to enable deconvolution of the merged t-SNE map into individual maps specific to each donor. Following dimensionality reduction, coordinates for each t-SNE dimension (i.e., t-SNE1 and t-SNE2) were determined for every cell and were integrated into a new appended fcs file as novel parameters.

**Figure 2:**
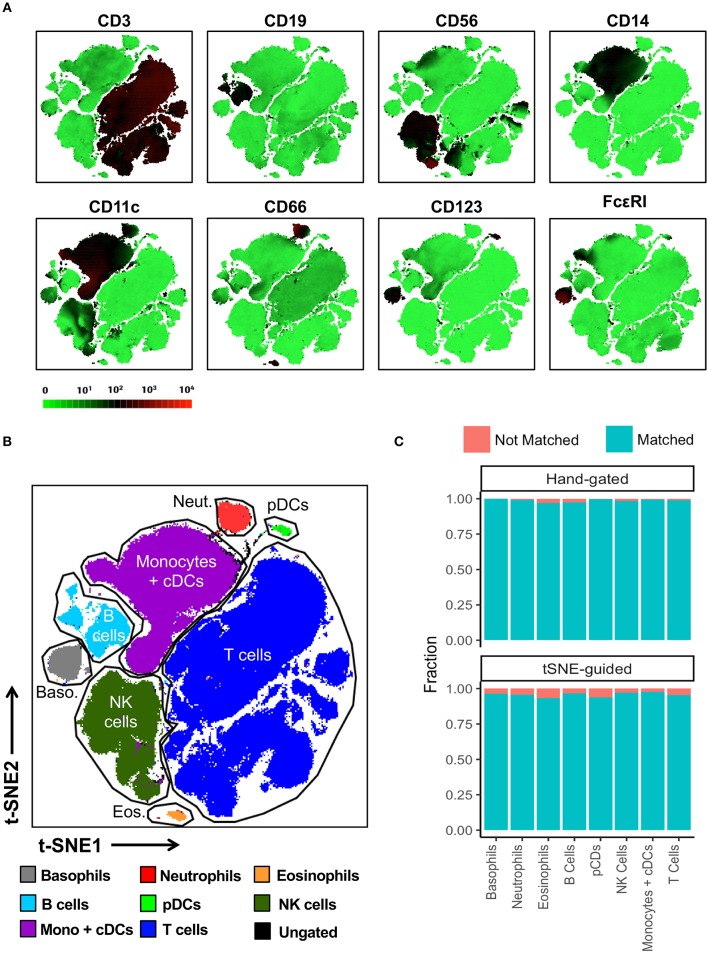
t-SNE-guided manual gating analysis of general immune lineages. **(A)** 10 healthy donor PBMC samples were merged to create a single t-SNE map with the signal strength of key phenotypic markers defining specific cellular lineages expressed with a green-black-red continuous color scale. t-SNE analysis was performed using 1,000 iterations, a perplexity of 30, a trade-off θ of 0.5, and all 38 of the phenotypic markers listed in Table S1. **(B)** Cell populations defined by the manual gating strategy in Figure 1 were projected onto t-SNE space and assigned specific colors. **(C)** The level of overlap or matching between conventional and t-SNE-guided manual gating analyses was calculated for every general cellular lineage. See Materials and Methods and Figure S5 for details on how the level of matching between analytical strategies was calculated. The proportion of cells that were not matched is shown in red while the proportion of cells that were matched is shown in blue.

In contrast to conventional sequential biaxial plot-based analysis, t-SNE analysis generates a single map in which the complex multi-dimensional geometric relationships between single cells are represented in a two-dimensional space. A third dimension using a color-based representation of the expression levels of a single parameter is then used in order to facilitate the identification of specific cellular lineages (10) (Figure 2A). Islands of cells can usually be deciphered on the t-SNE map, which often uniformly express lineage-specific markers such as CD3, CD19, and CD14. *A priori* knowledge of immunophenotyping can subsequently be applied to a series of these single-parameter maps in order to facilitate the supervised annotation of different cellular lineages. In this manner, on the global t-SNE map, 8 general cell populations were manually gated in t-SNE space and putatively identified as: basophils, neutrophils, eosinophils, plasmacytoid dendritic cells (pDCs), NK cells, monocytes and conventional dendritic cells (cDCs), B cells, and T cells. A small fraction of cells (0.24% of total) was left unidentified.

The overlap between cell populations identified via conventional and t-SNE-guided gating was qualitatively assessed by projecting hand-gated cell populations onto t-SNE space (Figure 2B). This approach showed minimal intermingling of cell populations between the different islands of cells stratified by dimensionality reduction. Subsequently, more quantitative methods to assess the relationship between conventional and t-SNE-guided gating were also applied both on a population level as well as single-cell level. Specifically, the frequencies (as % of total CD45+ cells) of each general cell population for each of the individual ten donors and the aggregated data (11 total data points) were determined and correlated between each analysis method (Figure S2A). This population-based analysis was highly correlated between both methods. The reproducibility of t-SNE-guided analysis across multiple independent t-SNE runs was also evaluated (Figure S3A). Due to the stochastic nature of t-SNE, the same cell populations fell in different parts of each map; however, the overall quantitation of the 8 general lineages was nearly identical between different runs (Figure S3B). A comparison of these analytical approaches also showed high correlation in the analysis of a 6-parameter flow cytometry data set demonstrating that high dimensional data is not required for separating distinct cell population in t-SNE space (Figure S4).

In order to assess overlap between conventional and t-SNE-guided analysis at the single-cell level, the cells that were captured by either analytical method for the aggregated donor data were compared for each cell subset. The fraction of cells captured by conventional gating that overlapped with those captured by t-SNE-guided gating were calculated to quantify the sensitivity of the t-SNE-guided gating method for replicating results from the conventional analysis. Analogously, the fraction of cells captured by t-SNE-guided gating matching those in the hand-gated population was also calculated (see Materials and Methods and Figure S5 for more details). Figure 2C shows that for the 8 general cell populations identified, 97% or more of the cells that were conventionally hand-gated were matched with the t-SNE-guided analysis. Cell populations identified based on t-SNE stratification were matched with hand-gated populations at slightly lower levels but still above 93% matching for these 8 populations. Similar results were obtained for manually segregating general T cell lineages in t-SNE space based on CD3, CD4, CD8, γδ TCR, Vα7.2 TCR, and CD161 expression (Figures S6, S2B).

### Comparison of Conventional and t-SNE-guided Manual Analysis Across Immune Cell Subsets

**Figure 3:**
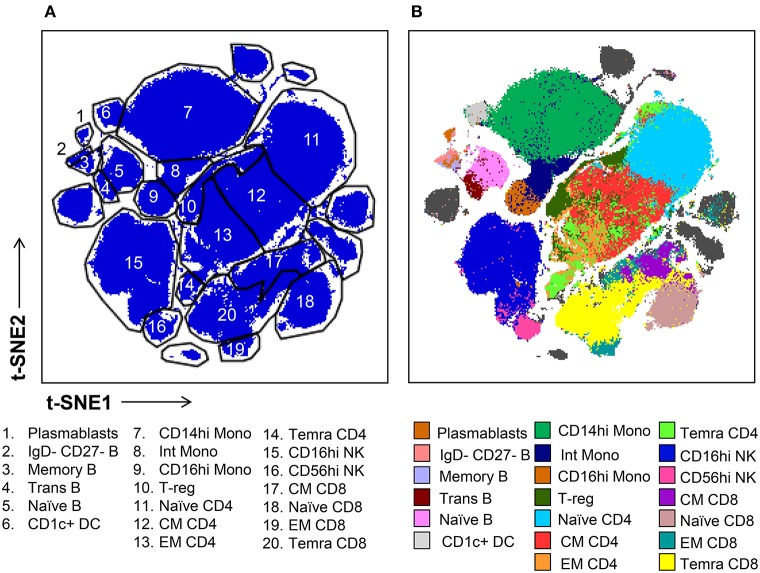
t-SNE-guided manual gating analysis of immune cell subsets. **(A)** Immune cell subsets were identified and manually gated in t-SNE space based on the signal intensity of the phenotypic markers shown in Figure S7A in order to correspond to the subsets defined in Figure 1. **(B)** Cell subsets defined by conventional manual analysis were projected into t-SNE space and assigned different colors.

Subsequently, we evaluated the ability of t-SNE mapping to stratify the deeper subsets of lymphocytes, monocytes, and mDCs which we identified via conventional gating in Figure 1. Specifically, the differential expression of the markers shown in Figure S7 were used to manually gate these general populations into sub lineages. Importantly, in contrast to the t-SNE-guided gating used to identify more general cell lineages, which leveraged the intrinsic topography of clearly separated cellular islands, for some subsets clear separation in t-SNE space was not observed. Thus, manual gates were drawn based on marker expression levels (analogous to the approach used in conventional gating in Figure 1) in the absence of topographic features that informed more objective boundaries (Figure 3A). Figure 3B demonstrates that certain subsets, especially central and effector memory T cell subsets, defined by conventional hand-gating were often commingled in t-SNE space.

**Figure 4:**
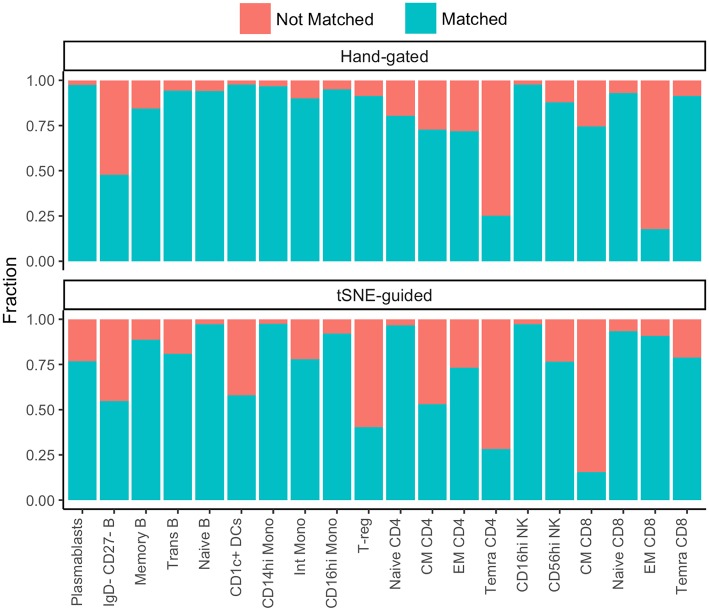
Correspondence between conventional and t-SNE-guided manual gating analyses for immune cell subsets. The level of overlap or matching between conventional and t-SNE-guided manual gating analyses was calculated for every general cellular lineage as in Figure 2C. The proportion of cells that were not matched is shown in red while the proportion of cells that were matched is shown in blue.

While some cell subpopulations were well matched (>90%) by both manual and t-SNE-guided gating others were only matched at low levels (<30%) (Figure 4). Naïve lymphocytes were matched most concordantly (>80%) most likely due to redundancy between markers specific to the naïve state such as CD45RA, CCR7, CD27, and IgD. In contrast, central and effector memory T cell subsets defined even in high dimensional space by the expression of continuous markers were not well matched. Overall, when t-SNE map topography inherently defined discrete boundaries between cellular islands there was a higher likelihood for overlap between both gating approaches.

### Isolated t-SNE Analysis of the CD4 T Cell Lineage

**Figure 5:**
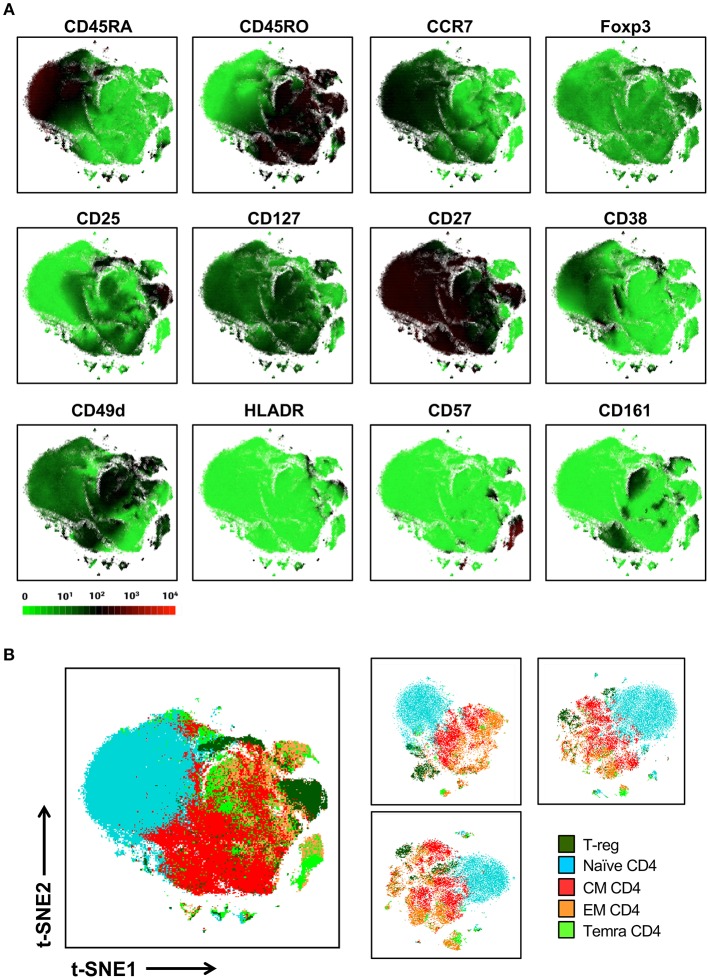
Local t-SNE mapping of only the CD4 T cell lineage does not clearly separate memory subsets defined by conventional manual analysis. **(A)** 250,000 CD4 T cells were extracted from the 10-donor data set and were reanalyzed using t-SNE as an isolated lineage using the entire panel as in Figure 2. Signal intensity for individual markers involved in defining various T cell subsets are shown. **(B)** Projection of hand-gated CD4 T cell subsets from 10 donor data set into t-SNE space (left panel). CD4 T cell t-SNE maps were independently generated for 3 individual donors (25,000 cells per donor right panel).

We next sought to determine whether t-SNE mapping of a single lineage that exhibited interspersion between manually defined canonical subsets in our global t-SNE map, could be better stratified with more discrete topographies when mapped as an individual lineage (Figure 5). To address this question, we isolated only the CD4+ T cell lineage (25,0000 cells from each of 10 donors) and ran t-SNE on this general population alone (with the entire 38 marker panel). Figure 5A shows the expression levels of markers relevant to the CD4 T cell lineage. While this approach led to more separation of minor T cell islands which became more distant from the main population in the CD4 T cell restricted analysis, it did not achieve clear resolution between general CD4+ T cell subsets other than the naïve subset (Figure 5B, left panel).

We hypothesized that parameters that were common to all CD4 T cells such as CD3 and CD4 were potentially restricting the ability of t-SNE to segregate CD4 T cell subsets. We compared t-SNE analysis of the CD4 T cell lineage with and without including markers either universally present or absent within this lineage and found little effect of constraining the markers used by t-SNE on the segregation of CD4 T cell subsets (data not shown).

Alternatively, it is possible that our inability to clearly delineate these populations in t-SNE space was the result of merging of different donors within a single map. This is because the conventional hand-gating performed on the merged file might not have been sufficiently tailored to the individual differences in marker expression which could vary between donors for either technical or biological reasons. To address this question t-SNE analysis was performed on individual donors with manual gating being tailored to each individual's particular expression patterns of CD25, Foxp3, CCR7, and CD45RA (Figure 5B, right panel). Again, we observed that while the naïve population is reproducibly segregated to one side of the map and shows little commingling with memory subsets, the other subsets are not clearly stratified within the t-SNE map. Thus, the lack of separation between memory T cell populations in t-SNE space was not due to the merger of different donors into a single t-SNE map.

Subsequently, we asked whether the application of a variety of clustering algorithms [Phenograph (16), DensVM (17), and FlowSOM (18)] could at least qualitatively stratify the 5 specific hand-gated T-cell populations we projected onto t-SNE space in Figure 5B; Figure S8. Naïve T-cells and a subset of T-regulatory cells were captured by one or a few clusters by each algorithm in a manner that was congruent with t-SNE map topography as well as manual hand gating. In contrast, the 3 other subsets of CD4 memory T-cells were not as clearly related to projected hand gates.

To further examine the effect of continuous markers on separation of phenotypes in t-SNE space, synthetic datasets containing two markers, M1 and M2 were created. Marker expressions for these channels were sampled from distributions with varying levels of continuity, including discrete bimodal, continuous bimodal, and unimodal (Figure S9). This was achieved by sampling two normal distributions with fixed mean values at negative and positive expression and variable standard deviation values to tune the level of continuity across these two levels. Dimensionality reduction using t-SNE was applied to these datasets to generate 2-dimensional projection of the data onto the t-SNE space. We observed that in the absence of a marker with discrete distribution, t-SNE is unable to fully resolve populations with continuous marker expression. This suggests that the distribution of marker expression is a contributing factor in the separation or intermingling of cells in t-SNE space.

### Modulation of t-SNE Parameters Does Not Fully Separate Cell Populations Defined by Continuous Variables

The pre-specification of iteration number, perplexity, and trade-off θ can potentially impact cell stratification in t-SNE space and we qualitatively assessed whether the modulation of these parameters could lead to more concordance between t-SNE-guided and manual gating (Figure S10). Increasing perplexity and iteration number and decreasing the trade-off θ resulted in more separation between distinct cellular lineages with discrete marker distribution (Figure S10A). In contrast, modulating these parameters only marginally impacted the analysis of memory T-cell populations with continuous marker distributions (Figure S10B). Barnes-hut approximation through introduction of trade-off parameter θ enables practical application of t-SNE on large datasets. Importantly, increasing the trade-off parameter θ to 0.8, thus moving away from exact t-SNE, resulted in the inability of t-SNE-based mapping to resolve pDCs, a rare cell population (Figure S10A).

## Discussion

The inability of t-SNE to clearly separate canonical memory T cell subsets defined by a priori knowledge is not an inherent defect in dimensionality reduction as an analytical approach. Our analysis revealed that the information in our data set was insufficient to fully separate central and effector memory T cell subsets using t-SNE most likely because the parameters we expected to fully differentiate memory T cells were continuously distributed. In contrast, conventional bivariate gating arbitrarily discretizes cell populations based on operator defined areas of low density between largely continuous underlying data distributions as in the case for CCR7, CD45RA, and CD45RO. In contrast, the t-SNE maps we examined did not arbitrarily separate cell populations with similar patterns and levels of cell surface marker expression. This does not mean that these observations call into question the existence of the central and effector memory T cell subsets, but that our data set did not provide sufficient information to clearly differentiate memory T cell populations in t-SNE space. We found this to be true both in the context of a global as well as local t-SNE map in which only the CD4 T cell lineage was visualized (Figure 5). Lineage extraction and local t-SNE map generation was also performed for the monocyte & dendritic cell lineage (data not shown). We found that in global t-SNE maps dendritic cells typically formed a peninsular structure jutting out from the “mainland” of classical CD14hi monocytes, and in at least one local map we generated, DCs did achieve full separation from the major monocyte population (data not shown). Thus, finer resolution can be achieved in a local map; however, this did not seem to significantly alter the interpretation of the topography of a global t-SNE map. A hierarchical approach to t-SNE has been described (26), and the field should continue to evaluate the relative value of global t-SNE analyses vs. local analyses in which only specific lineages are isolated and visualized.

It is becoming convention for dimensionality reduction and clustering algorithms to be applied in tandem to single-cell data sets with populations defined by clustering algorithms being projected onto t-SNE space (16). This approach is undoubtedly more objective and reproducible than performing t-SNE-guided manual gating to segregate populations with continuous marker distributions, which recapitulates the “original sin” of manual hand gating.

It is currently unclear whether the use of t-SNE derived coordinates as part of a clustering approach is preferable to using these tools independently. Distance in t-SNE space should not be overinterpreted since distinct cell populations that are close in one t-SNE analysis can be distant in a second analysis of the same data set (Figure S3). Thus, it is questionable whether t-SNE coordinates should be included in defining cellular clusters. Regardless, a comparison of how clusters projected onto t-SNE space compare to conventional manual analysis is outside the scope of these studies and would be redundant with publications that have already performed this comparison without t-SNE visualization (6).

Going forward, an essential question for visualizing single-cell data with both dimensionality reduction and clustering is whether these approaches can deliver messages that are contradictory about the same data set. For example, what does an analyst conclude if a t-SNE map does not display distinct and well separated cellular islands but a clustering algorithm applied to the map parses out a variety of clusters that are only continuously differentiated in t-SNE space (as in Figure S8)? The biological relevance of poorly differentiated clusters should be assessed with orthogonal functional assays or additional markers providing more robust phenotypic stratification.

The application of t-SNE to single cell analysis probably provides the most value in efforts to survey the cellular heterogeneity of complex tissues and to characterize novel or poorly defined cell populations. In these studies, we tested the ability of the t-SNE approach to stratify familiar cell populations in an extremely well characterized sample matrix. If our studies had identified profound discrepancies between our “ground truth” conventional analysis of canonical subsets and the t-SNE-guided analysis, we may have questioned whether this approach is appropriate for the characterization of poorly defined cell populations or whether our ground truth assumptions were incorrect. Instead, a high degree of overlap in the general cellular lineages defined by these approaches was found, and the identified discrepancies led us to revisit the logic of subjectively discretizing continuous variables rather than the validity of dimensionality reduction.

## Ethics Statement

This study was carried out in accordance with the recommendations of the Western Institutional Review Board (WIRB) as part of the Genentech Employee Blood donation program with written informed consent from all subjects. All subjects gave written informed consent in accordance with the Declaration of Helsinki. The protocol was approved by the WIRB.

## Author Contributions

WO oversaw research, designed experiments, and wrote manuscript. ST analyzed data, generated t-SNE plots, and adapted an R based t-SNE package created by CB who also aided in these activities. AA-Y, CT, MN, and SL generated and analyzed data. CG and WM oversaw research and aided in manuscript preparation.

### Conflict of Interest Statement

The authors declare that this study received funding from Genentech. All authors are employees of Genentech. The funder had no role in study design, data collection and analysis, decision to publish, or preparation of the manuscript. The authors declare that the research was conducted in the absence of any commercial or financial relationships that could be construed as a potential conflict of interest.

## Supplementary Material

Figure S1 Overview of t-SNE-guided analysis strategy. PBMCs were prepared from 10 healthy donors and analyzed via mass cytometry. Live singlet cells were manually gated using FlowJo software and individually exported as fcs files. Following file merger t-SNE analysis was conducted as described in Materials and Methods. A new fcs file was written for the merged data set as well as each individual donor with appended t-SNE parameters, which was then manually gated in t-SNE space using Flowjo. t-SNE-guided gates were then copied from the merged data to each individual donor.

Figure S2 Correlation of cell subset frequencies. Cell frequencies were obtained from conventional and t-SNE-guided gating of individual donors and the aggregated data for **(A)** general immune lineages, **(B)** general T cell lineages, and **(C)** immune cell subsets.

Figure S3 The stochastic nature of the t-SNE algorithm results in different maps in multiple runs. **(A)** Hand-gated subsets were projected onto t-SNE maps generated from 3 independent t-SNE runs. **(B)** Cell frequencies of general immune subsets obtained from t-SNE-guided gating of multiple runs are not affected by the topological changes of the maps.

Figure S4 t-SNE analysis of PBMCs analyzed by a 6-parameter flow cytometry assay. **(A)** t-SNE map overlaid with expression of phenotypic markers. **(B)** Cell populations defined by the manual gating strategy in Figure 1 were projected onto t-SNE space and assigned specific colors. **(C)** Correlation of cell population frequencies obtained from conventional and t-SNE-guided gating.

Figure S5 Comparison of hand-gated vs. t-SNE-guided analysis at the single-cell level. Concordance between the two gating strategies was quantified by the fraction of cells present in both gates. For every cell population A_h_ represents the number of cells included in hand-drawn conventional gate and excluded from the t-SNE-guided gate, A_t_ is the number of cells included in t-SNE-guided gate and excluded from the hand-gated conventional gate and B is the number of cells that were present in both gating strategies.

Figure S6 t-SNE-guided manual gating analysis of T cell lineages. **(A)** t-SNE map overlaid with signal intensities of key phenotypic markers. **(B)** Cell populations defined by the manual gating strategy in Figure 1 were projected onto t-SNE space and assigned specific colors. **(C)** Level of overlap between conventional and t-SNE-guided manual gating is shown in blue.

Figure S7 Additional t-SNE maps of aggregated 10-donor data. **(A)** Markers used for t-SNE-guided analysis of immune cells subsets. **(B)** Additional markers included in the generation of the t-SNE map but not for t-SNE-guided subsetting.

Figure S8 Computationally defined CD4 T-cell clusters projected onto t-SNE space. Data from Figure 5 were clustered with 3 different computational algorithms: Phenograph **(A)**, DensVM **(B)**, and FlowSOM with k = 20 **(C)**.

Figure S9 Impact of marker expression distribution on separation of subsets in t-SNE space in synthetic datasets with 1,000 data points and 2 markers. **(A)** Markers have a bimodal and discrete distribution. Data points are fully separated in the t-SNE space. **(B)** Markers have a bimodal, yet continuous distribution. Although some structure is seen on the t-SNE map that distinguishes double positive, double negative, M1hi M2lo and M1lo M2hi groups, the data subsets are not fully separated. **(C)** Markers have a unimodal distribution. t-SNE is unable to resolve any subsets. t-SNE was run with perplexity parameter of 30 and trade-off θ of 0.5.

Figure S10 Evaluation of the impact of algorithm parameters (iteration number, perplexity, and trade-off θ values) on t-SNE. 50,000 cells were analyzed using iteration number of 1,000 and 10,000, perplexity of 5, 30, and 100, and trade off θ values of 0.2, 0.5, and 0.8 for all PBMC lineages **(A)** and only CD4 T cells **(B)**.

Table S1 List of metal conjugated mAbs and their targets used in the 38-parameter cytometry panel.

## References

[1] Mair F Hartmann FJ Mrdjen D Tosevski V Krieg C Becher B. The end of gating? An introduction to automated analysis of high dimensional cytometry data. Eur J Immunol. (2015) 46:34–43. 10.1002/eji.201545774 26548301

[2] Finak G Frelinger J Jiang W Newell EW Ramey J Davis MM. OpenCyto: an open source infrastructure for scalable, robust, reproducible, and automated, end-to-end flow cytometry data analysis. PLoS Comput Biol. (2014) 10:e1003806. 10.1371/journal.pcbi.1003806 25167361 PMC4148203

[3] Finak G Langweiler M Jaimes M Malek M Taghiyar J Korin Y Standardizing flow cytometry immunophenotyping analysis from the human immunophenotyping consortium. Nat Publish Group. (2016) 2016:1–11. 10.1038/srep20686 PMC4748244 26861911

[4] Saeys Y Gassen SV Lambrecht BN. Computational flow cytometry: helping to make sense of high-dimensional immunology data. Nat Rev Immunol. (2016) 16:449–62. 10.1038/nri.2016.56 27320317

[5] Weber LM Robinson MD. Comparison of clustering methods for high-dimensional single-cell flow and mass cytometry data. Cytometry. (2016) 89:1084–96. 10.1002/cyto.a.23030 27992111

[6] Aghaeepour N Finak G Dougall D Khodabakhshi AH Mah P Obermoser G. Critical assessment of automated flow cytometry data analysis techniques. Nat Methods. (2013) 10:228–38. 10.1038/nmeth.2365 23396282 PMC3906045

[7] Becht E Dutertre C-A Kwok IWH Ng LG Ginhoux F Newell EW Evaluation of UMAP as an alternative to t-SNE for single-cell data. bioRxiv. (2018) 2018:1–10. 10.1101/298430

[8] Konstorum A Vidal E Jekel N Laubenbacher R Comparative analysis of linear and nonlinear dimension reduction techniques on mass cytometry data. bioRxiv. (2018) 2018:1–15. 10.1101/273862

[9] Van Der Maaten L Res GHJML Visualizing high-dimensional data using t-SNE. J Mach Learn Res. (2008) 9:2579–605. Available online at: http://www.jmlr.org/papers/v9/vandermaaten08a.html

[10] Amir E-AD Davis KL Tadmor MD Simonds EF Levine JH Bendall SC viSNE enables visualization of high dimensional single-cell data and reveals phenotypic heterogeneity of leukemia. Nat Biotechnol. (2013) 31:545–52. 10.1038/nbt.2594 23685480 PMC4076922

[11] Guilliams M Dutertre C-A Scott CL McGovern N Sichien D Chakarov S. Unsupervised high-dimensional analysis aligns dendritic cells across tissues and species. Immuni. (2016) 45:669–84. 10.1016/j.immuni.2016.08.015 27637149 PMC5040826

[12] Wong MT Ong DEH Lim FSH Teng KWW McGovern N Narayanan S. A high-dimensional atlas of human T cell diversity reveals tissue-specific trafficking and cytokine signatures. Immuni. (2016) 45:442–56. 10.1016/j.immuni.2016.07.007 27521270

[13] Hahne F LeMeur N Brinkman RR Ellis B Haaland P Sarkar D. flowCore: a Bioconductor package for high throughput flow cytometry. BMC Bioinformatics. (2009) 10:106. 10.1186/1471-2105-10-106 19358741 PMC2684747

[14] Software JKC Rtsne: T-Distributed Stochastic Neighbor Embedding using Barnes-Hut Implementation (R package version 0.10) (2015).

[15] Van Der Maaten Accelerating t-SNE using tree-based algorithms. J Mach Learn Res L. (2014) 15:3221–45. Available online at: http://jmlr.org/papers/v15/vandermaaten14a.html

[16] Levine JH Simonds EF Bendall SC Davis KL Amir E-AD Tadmor MD Data-driven phenotypic dissection of AML reveals progenitor-like cells that correlate with prognosis. Cell. (2015) 2015:47 10.1016/j.cell.2015.05.047 PMC4508757 26095251

[17] Vazquez J Chavarria M Li Y Lopez GE Stanic AK. Computational flow cytometry analysis reveals a unique immune signature of the human maternal-fetal interface. Am J Reprod Immunol. (2017) 79:e12774–15. 10.1111/aji.12774 29030900 PMC5725254

[18] Van Gassen S Callebaut B Van Helden MJ Lambrecht BN Demeester P Dhaene T FlowSOM: using self-organizing maps for visualization and interpretation of cytometry data. Cytometry. (2015) 2015:22625 10.1002/cyto.a.22625 25573116

[19] Chen H Lau MC Wong MT Newell EW Poidinger M Chen J. Cytofkit: a bioconductor package for an integrated mass cytometry data analysis pipeline. PLoS Comput Biol. (2016) 12:e1005112–17. 10.1371/journal.pcbi.1005112 27662185 PMC5035035

[20] Thurau AM Schylz U Wolf V Krug N Schauer U. Identification of eosinophils by flow cytometry. Cytometry. (1996) 23:150–8. 10.1002/(SICI)1097-0320(19960201)23:2<150::AID-CYTO8>3.0.CO;2-O 8742174

[21] Mahnke YD Beddall MH Roederer M OMIP-013: differentiation of human T-cells. Cytometry. (2012) 81A:935–6. 10.1002/cyto.a.22201 23027685

[22] Fergusson JR Smith KE Fleming VM Rajoriya N Newell EW Simmons R. CD161 defines a transcriptional and functional phenotype across distinct human T cell lineages. Cell Rep. (2014) 9:1075–88. 10.1016/j.celrep.2014.09.045 25437561 PMC4250839

[23] Wei C Jung J Sanz I OMIP-003: phenotypic analysis of human memory B cells. Cytometry. (2011) 79A:894–6. 10.1002/cyto.a.21112 PMC3199331 21796774

[24] Mahnke YD Beddall MH Roederer M. OMIP-029: human NK-cell phenotypization. Cytometry. (2015) 87:986–8. 10.1002/cyto.a.22728 26228006

[25] O'Gorman WE Hsieh EWY Savig ES Gherardini PF Hernandez JD Hansmann L. Single-cell systems-level analysis of human Toll-like receptor activation defines a chemokine signature in patients with systemic lupus erythematosus. J Allergy Clin Immunol. (2015) 136:1326–36. 10.1016/j.jaci.2015.04.008 26037552 PMC4640970

[26] Unen V Höllt T Pezzotti N Li N Reinders MJT Eisemann E Visual analysis of mass cytometry data by hierarchical stochastic neighbour embedding reveals rare cell types. Nat Commun. (2017) 2017:1–9. 10.1038/s41467-017-01689-9 PMC5700955 29170529

