# Supplementary material for: Quantitative Comparison of Conventional and t-SNE-guided Gating Analyses
Source: Front Immunol. 2019 Jun 5;10:1194. doi: 10.3389/fimmu.2019.01194 (PMC6560168; doi:10.3389/fimmu.2019.01194)
Supplement: Figure S1 — Overview of t-SNE-guided analysis strategy. PBMCs were prepared from 10 healthy donors and analyzed via mass cytometry. Live singlet cells were manually gated using FlowJo software and individually exported as fcs files. Following file merger t-SNE analysis was conducted as described in Materials and Methods. A new fcs file was written for the merged data set as well as each individual donor with appended t-SNE parameters, which was then manually gated in t-SNE space using Flowjo. t-SNE-guided gates were then copied from the merged data to each individual donor. [file Data_Sheet_1.pdf]

Table S1

Mass	Metal	Target	Source	Vendor	Clone
89	Y	CD45	Fluidigm		HI30
115	In	CD57	Custom	Biolegend	HCD57
140	Ce	EQ Beads			
141	Pr	CD49d	Fluidigm		9F10
142	Nd	CCR4	Custom	R&D	205410
143	Nd	CD127	Fluidigm		A019D5
144	Nd	Granzyme B	Custom	Biolegend	GB11
145	Nd	CD4	Fluidigm		RPA-T4
146	Nd	IgD	Fluidigm		IA6-2
147	Sm	CD7	Fluidigm		CD7-6B7
148	Nd	CD16	Fluidigm		3G8
149	Sm	Granzyme A	Custom	Biolegend	CB9
150	Nd	CD103	Custom	Biolegend	Ber-ACT8
151	Eu	CD123	Fluidigm		6H6
152	Sm	γδTCR	Custom	Life Technologies	SA6.E9
154	Sm	CD3	Fluidigm		UCHT1
155	Gd	CD27	Fluidigm		L128
156	Gd	CXCR3	Fluidigm		G025H7
157	Gd	CD19	Custom	Biolegend	HIB19
158	Gd	Vα7.2	Custom	Biolegend	3C10
159	Tb	CD11c	Fluidigm		Bu15
160	Gd	CD56	Custom	Miltenyi	REA196
161	Dy	CD66	Custom	BD	B1.1
162	Dy	Foxp3	Fluidigm		PCH101
163	Dy	CD20	Custom	Biolegend	2H7
164	Du	CD161	Fluidigm		HP-3G10
165	Ho	CD45RO	Fluidigm		UCHL1
166	Er	FcεRI	Custom	Biolegend	AER-37 (CRA-1)
167	Er	CCR7	Fluidigm		G043H7
168	Er	CD8	Fluidigm		SK1
169	Tm	CD25	Fluidigm		2A3
170	Er	CD45RA	Fluidigm		HI100
171	Yb	CD1c	Custom	Biolegend	L161
172	Yb	CD14	Custom	Biolegend	M5E2
173	Yb	PD-1	Custom	Biolegend	EH12.2H7
174	Yb	HLA-DR	Fluidigm		L243
175	Lu	Perforin	Fluidigm		B-D48
176	Yb	CD38	Custom	Biolegend	HIT2
191	Ir	Nucleic acid	Fluidigm		
192	Pt	Cisplatin	Fluidigm		
193	Ir	Nucleic acid	Fluidigm		
195	Pt	Cisplatin	Fluidigm		
209	Bi	CD11b	Fluidigm		ICRF-44

Figure S1

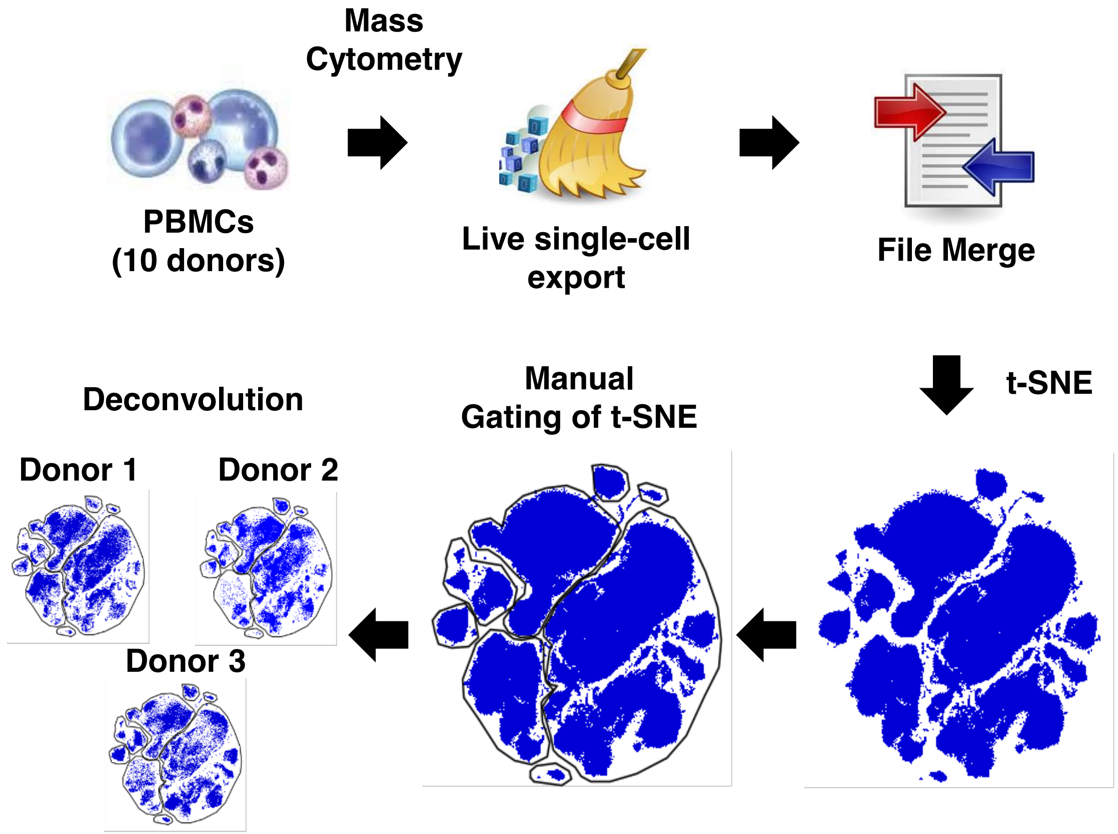

Mass
Cytometry
PBMCs
(10 donors)
Live single-cell
export
File Merge
t-SNE
Manual
Gating of t-SNE
Deconvolution
Donor 1
Donor 2
Donor 3

Figure S2

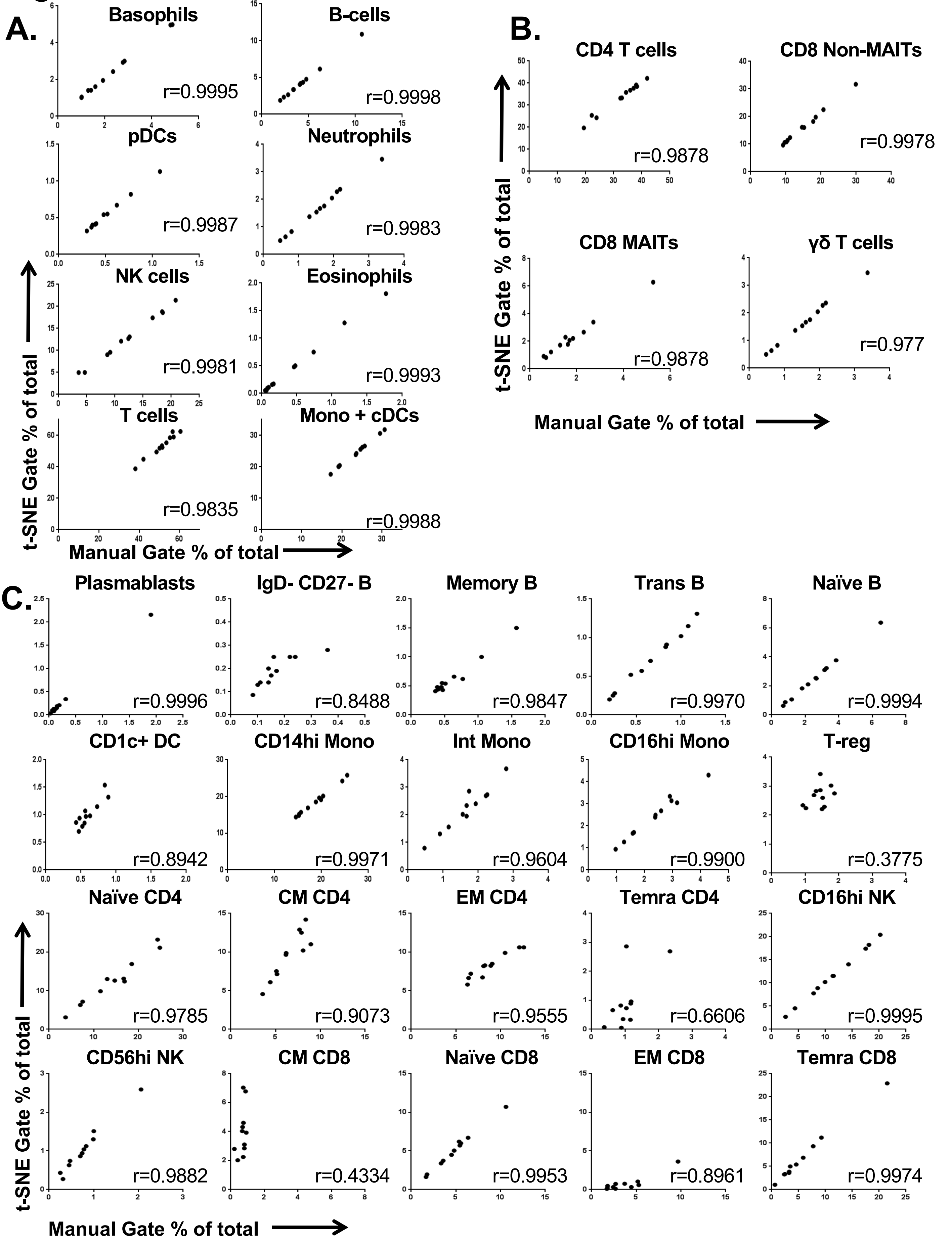
A.
Basophils
r=0.9995
B-cells
r=0.9998
pDCs
r=0.9987
Neutrophils
r=0.9983
NK cells
r=0.9981
Eosinophils
r=0.9993
T cells
r=0.9835
Mono + cDCs
r=0.9988
t-SNE Gate % of total
Manual Gate % of total
B.
CD4 T cells
r=0.9878
CD8 Non-MAITs
r=0.9978
CD8 MAITs
r=0.9878
γδ T cells
r=0.977
t-SNE Gate % of total
Manual Gate % of total
C.
Plasmablasts
r=0.9996
IgD- CD27- B
r=0.8488
Memory B
r=0.9847
Trans B
r=0.9970
Naïve B
r=0.9994
CD1c+ DC
r=0.8942
CD14hi Mono
r=0.9971
Int Mono
r=0.9604
CD16hi Mono
r=0.9900
T-reg
r=0.3775
Naïve CD4
r=0.9785
CM CD4
r=0.9073
EM CD4
r=0.9555
Temra CD4
r=0.6606
CD16hi NK
r=0.9995
CD56hi NK
r=0.9882
CM CD8
r=0.4334
Naïve CD8
r=0.9953
EM CD8
r=0.8961
Temra CD8
r=0.9974
t-SNE Gate % of total
Manual Gate % of total

Figure S3

A.

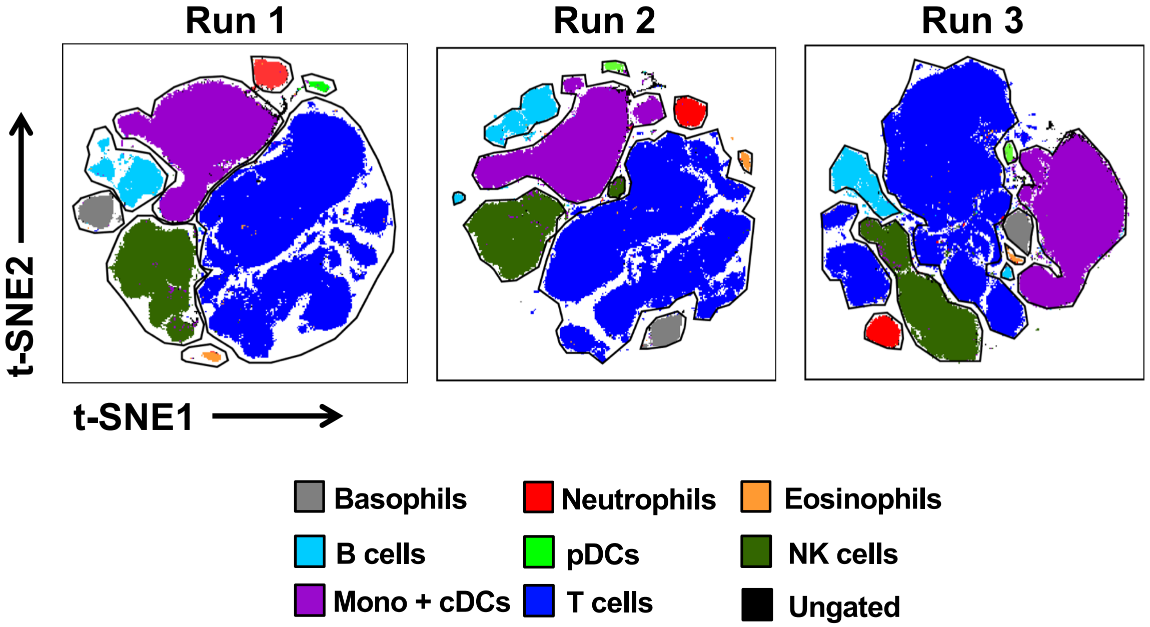
Run 1
Run 2
Run 3
t-SNE2
t-SNE1
Basophils
Neutrophils
Eosinophils
B cells
pDCs
NK cells
Mono + cDCs
T cells
Ungated

B.

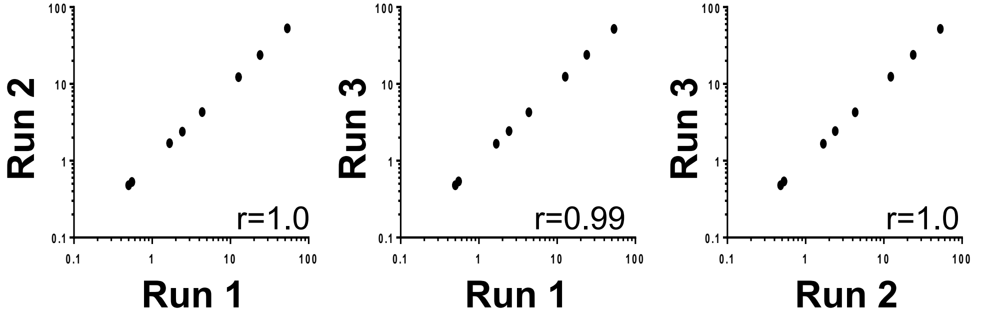
Run 2
Run 1
r=1.0
Run 3
Run 1
r=0.99
Run 3
Run 2
r=1.0
0.1
1
10
100

Figure S4

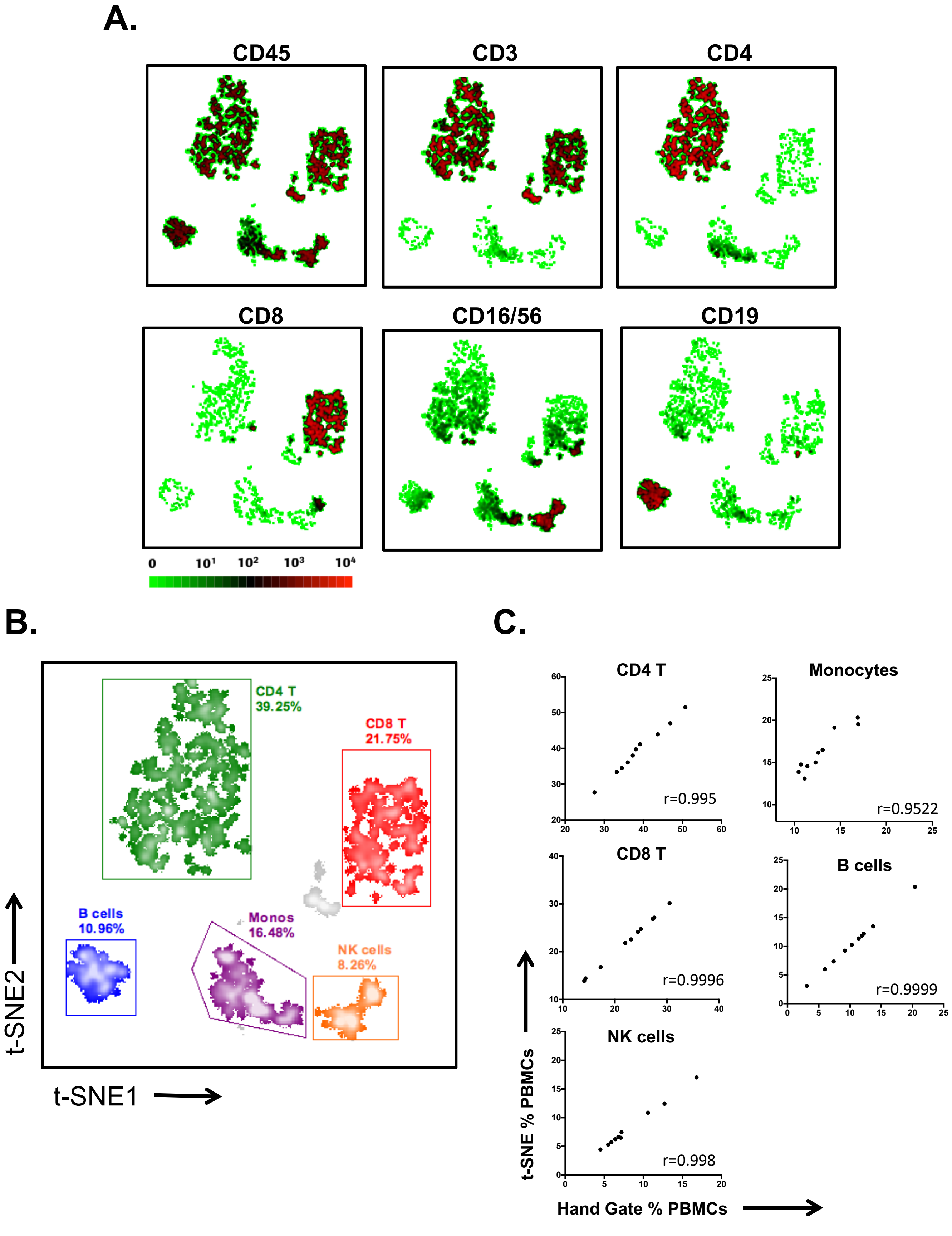
A.
CD45
CD3
CD4
CD8
CD16/56
CD19
0
10¹
10²
10³
10⁴
B.
CD4 T
39.25%
CD8 T
21.75%
B cells
10.96%
Monos
16.48%
NK cells
8.26%
t-SNE2
t-SNE1
C.
CD4 T
r=0.995
Monocytes
r=0.9522
CD8 T
r=0.9996
B cells
r=0.9999
NK cells
r=0.998
t-SNE % PBMCs
Hand Gate % PBMCs

Figure S5

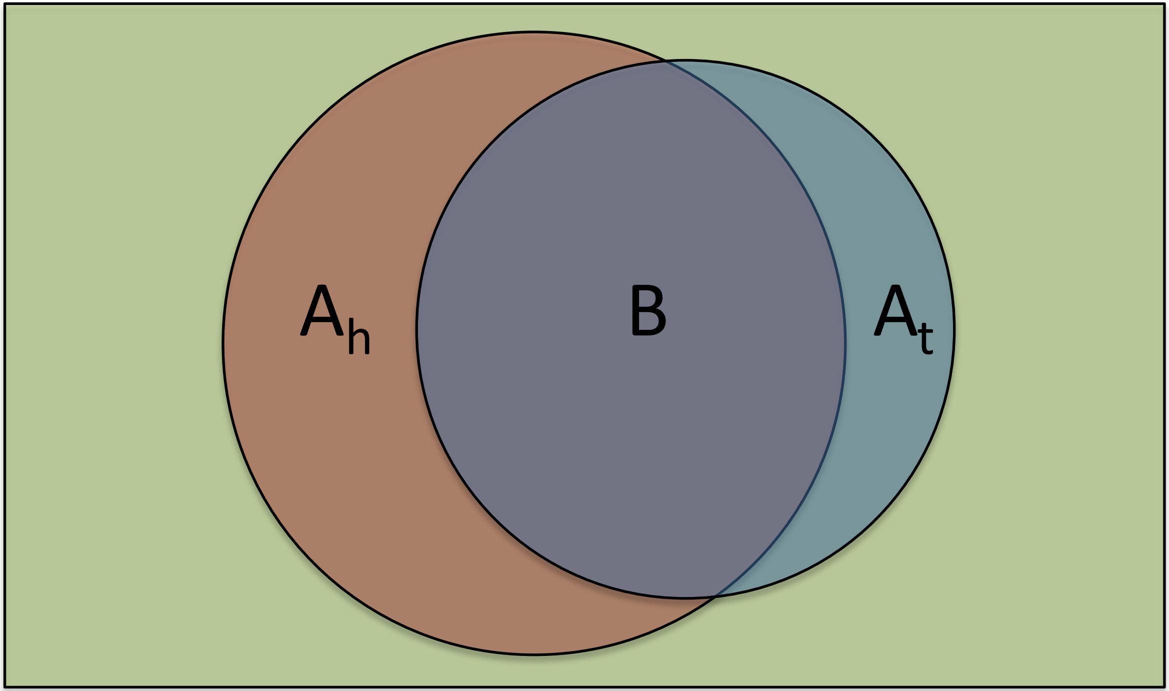

$\text{Matched}_{\text{hand-gated}} = B / (A_h + B)$

$\text{Matched}_{\text{tSNE-guided}} = B / (A_t + B)$

Figure S6

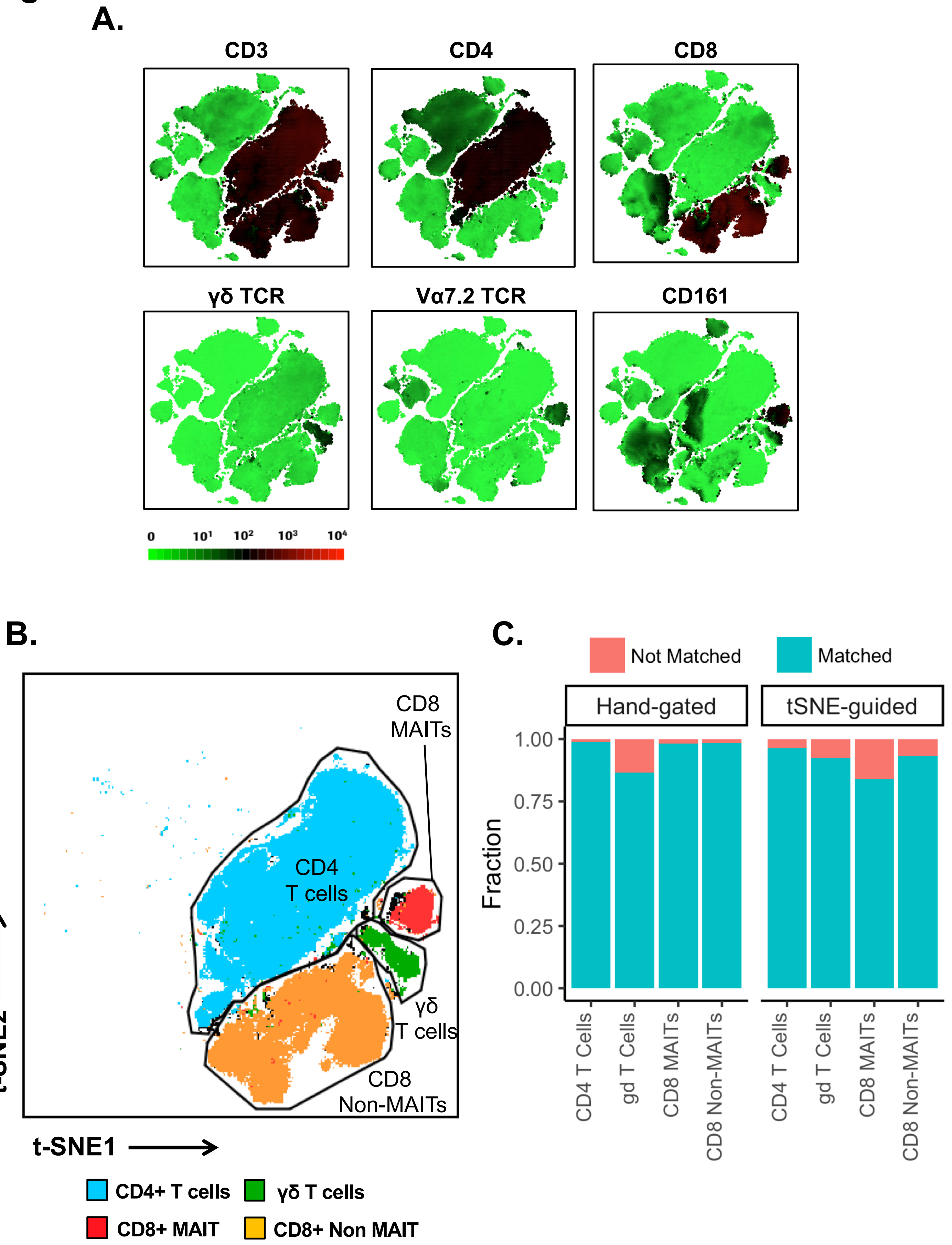

A.
CD3
CD4
CD8
γδ TCR
Vα7.2 TCR
CD161
0
10¹
10²
10³
10⁴
B.
CD8
MAITs
CD4
T cells
γδ
T cells
CD8
Non-MAITs
t-SNE2
t-SNE1
CD4+ T cells
γδ T cells
CD8+ MAIT
CD8+ Non MAIT
C.
Not Matched
Matched
Hand-gated
tSNE-guided
Fraction
1.00
0.75
0.50
0.25
0.00
CD4 T Cells
gd T Cells
CD8 MAITs
CD8 Non-MAITs

Figure S7

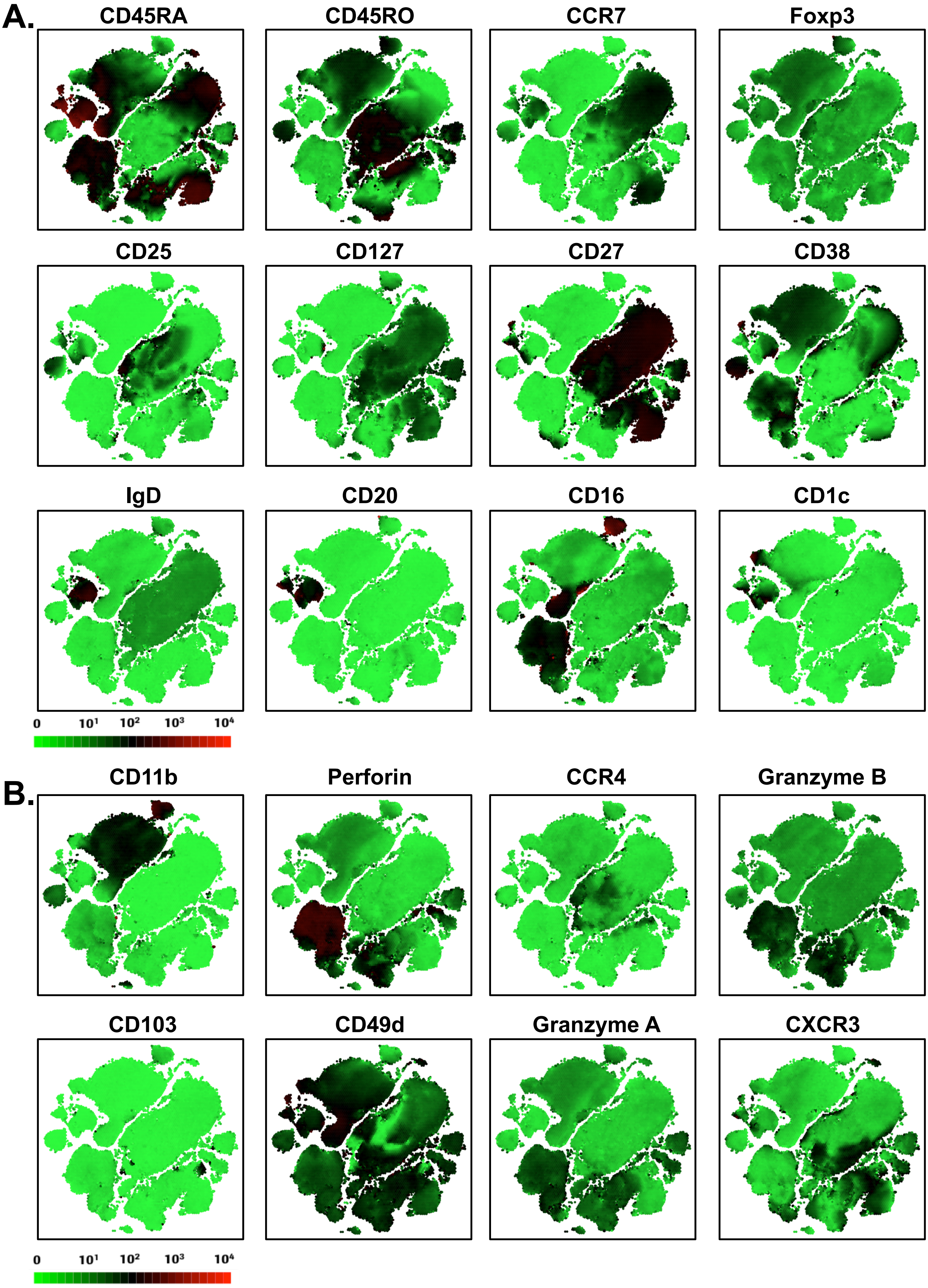
A.
CD45RA
CD45RO
CCR7
Foxp3
CD25
CD127
CD27
CD38
IgD
CD20
CD16
CD1c
0
10¹
10²
10³
10⁴
B.
CD11b
Perforin
CCR4
Granzyme B
CD103
CD49d
Granzyme A
CXCR3
0
10¹
10²
10³
10⁴

Figure S8

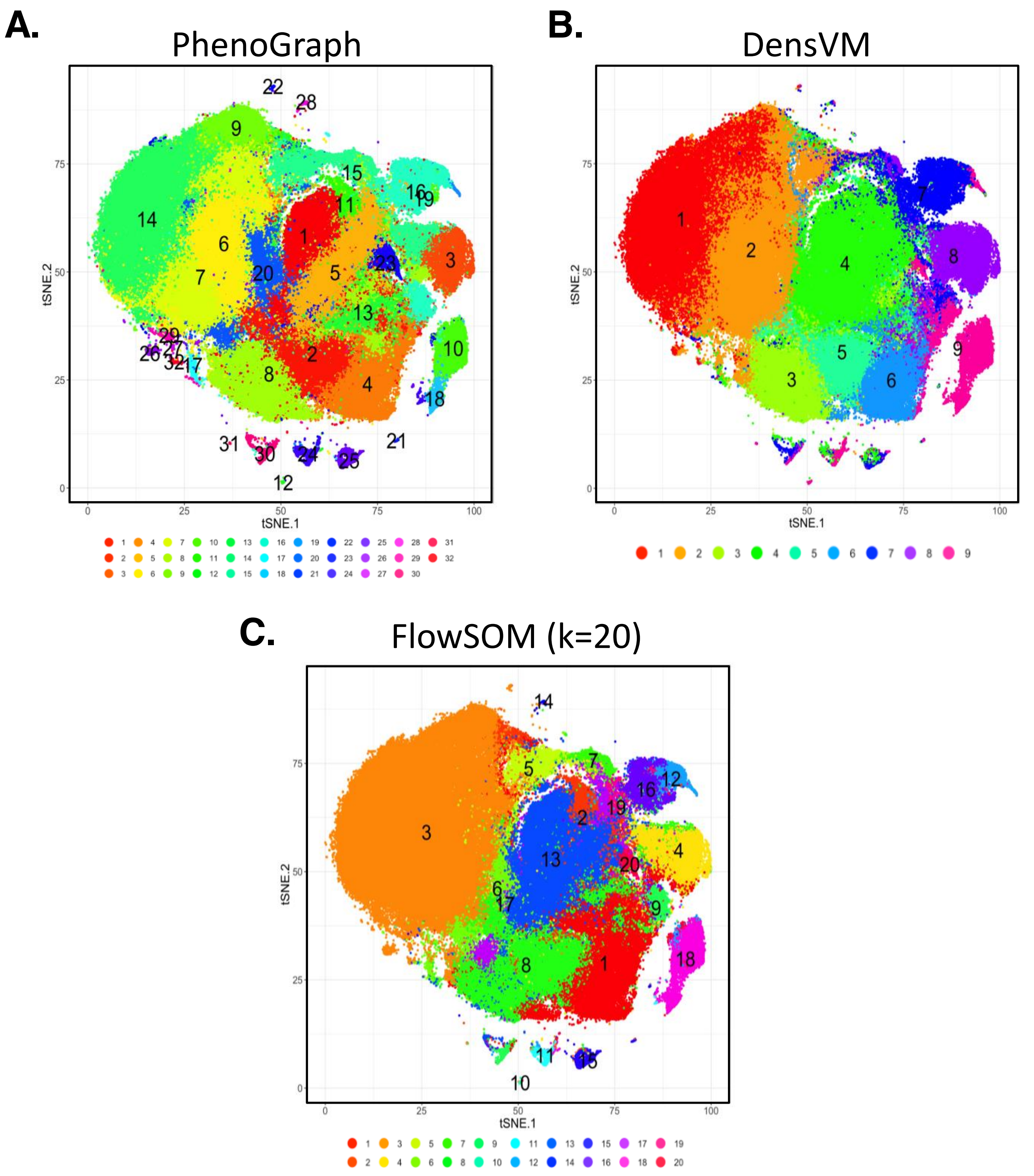

Figure S9

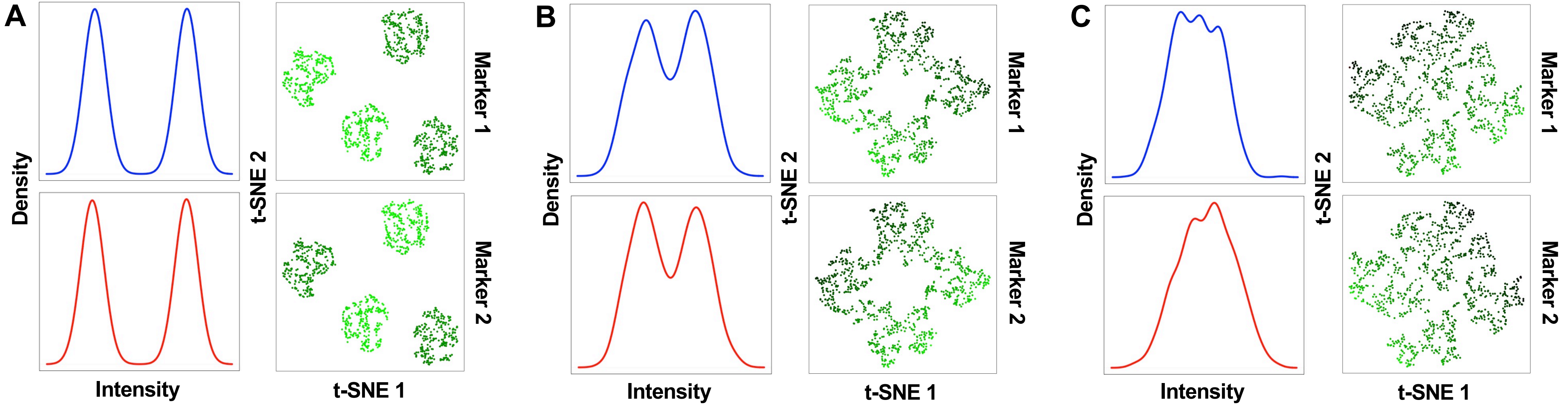

A
Density
Intensity
t-SNE 2
t-SNE 1
Marker 1
Marker 2
B
Density
Intensity
t-SNE 2
t-SNE 1
Marker 1
Marker 2
C
Density
Intensity
t-SNE 2
t-SNE 1
Marker 1
Marker 2

Figure S10

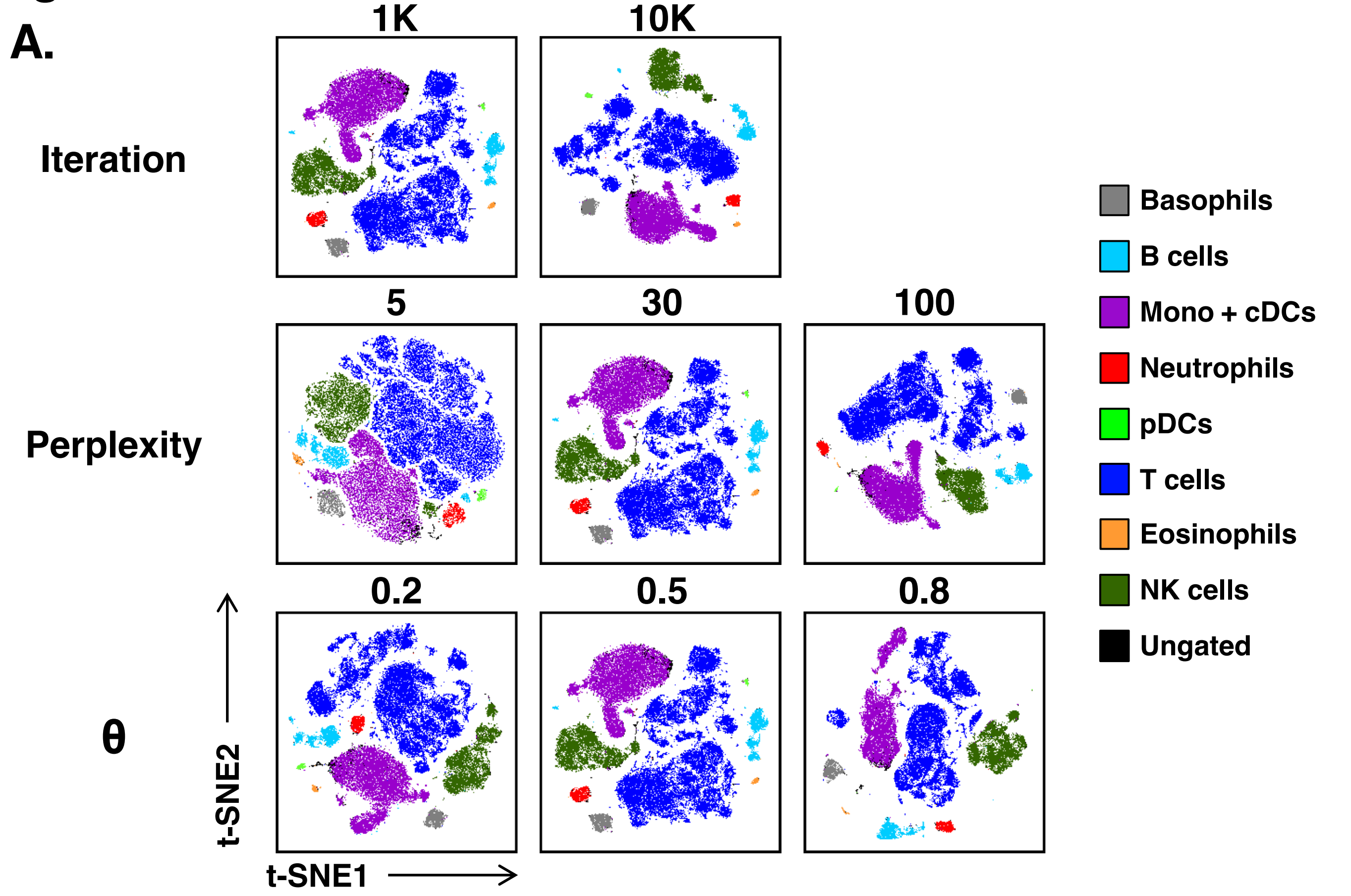
A.
1K
10K
Iteration
5
30
100
Perplexity
0.2
0.5
0.8
θ
t-SNE2
t-SNE1
Basophils
B cells
Mono + cDCs
Neutrophils
pDCs
T cells
Eosinophils
NK cells
Ungated

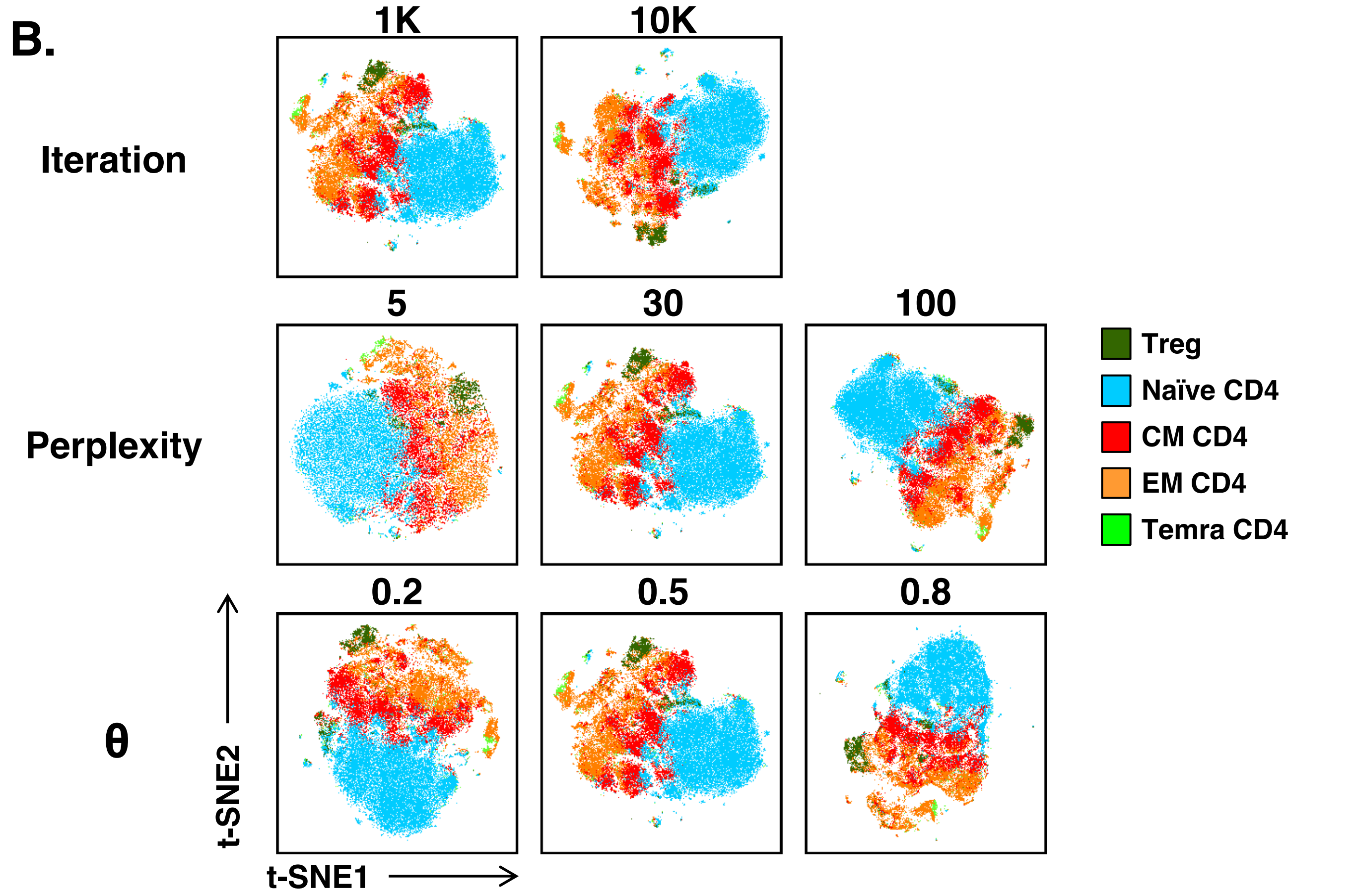
B.
1K
10K
Iteration
5
30
100
Perplexity
0.2
0.5
0.8
θ
t-SNE2
t-SNE1
Treg
Naïve CD4
CM CD4
EM CD4
Temra CD4
